# Mesenchymal Stem Cell Conditioning Promotes Rat Oligodendroglial Cell Maturation

**DOI:** 10.1371/journal.pone.0071814

**Published:** 2013-08-12

**Authors:** Janusz Joachim Jadasz, David Kremer, Peter Göttle, Nevena Tzekova, Julia Domke, Francisco J. Rivera, James Adjaye, Hans-Peter Hartung, Ludwig Aigner, Patrick Küry

**Affiliations:** 1 Department of Neurology, Medical Faculty, University of Düsseldorf, Düsseldorf, Germany; 2 Spinal Cord Injury and Tissue Regeneration Center Salzburg, Paracelsus Medical University, Salzburg, Austria; 3 Wellcome Trust and MRC Cambridge Stem Cell Institute & Department of Veterinary Medicine, University of Cambridge, Cambridge, United Kingdom; 4 Institute of Molecular Regenerative Medicine, Paracelsus Medical University, Salzburg, Austria; 5 Institute for Stem Cell Research and Regenerative Medicine, Medical Faculty, University of Düsseldorf, Düsseldorf, Germany; Hospital Nacional de Parapléjicos - SESCAM, Spain

## Abstract

Oligodendroglial progenitor/precursor cells (OPCs) represent the main cellular source for the generation of new myelinating oligodendrocytes in the adult central nervous system (CNS). In demyelinating diseases such as multiple sclerosis (MS) myelin repair activities based on recruitment, activation and differentiation of resident OPCs can be observed. However, the overall degree of successful remyelination is limited and the existence of an MS-derived anti-oligodendrogenic milieu prevents OPCs from contributing to myelin repair. It is therefore of considerable interest to understand oligodendroglial homeostasis and maturation processes in order to enable the development of remyelination therapies. Mesenchymal stem cells (MSC) have been shown to exert positive immunomodulatory effects, reduce demyelination, increase neuroprotection and to promote adult neural stem cell differentiation towards the oligodendroglial lineage. We here addressed whether MSC secreted factors can boost the OPC’s oligodendrogenic capacity in a myelin non-permissive environment. To this end, we analyzed cellular morphologies, expression and regulation of key factors involved in oligodendroglial fate and maturation of primary rat cells upon incubation with MSC-conditioned medium. This demonstrated that MSC-derived soluble factors promote and accelerate oligodendroglial differentiation, even under astrocytic endorsing conditions. Accelerated maturation resulted in elevated levels of myelin expression, reduced glial fibrillary acidic protein expression and was accompanied by downregulation of prominent inhibitory differentiation factors such as Id2 and Id4. We thus conclude that apart from their suggested application as potential anti-inflammatory and immunomodulatory MS treatment, these cells might also be exploited to support endogenous myelin repair activities.

## Introduction

Multiple sclerosis (MS) is the most common chronic inflammatory disease of the central nervous system (CNS) and a leading cause of fixed neurological disability of young adults in Western countries. MS is mediated by an immune response against myelin sheaths and myelin-producing cells, the oligodendrocytes. Degeneration and loss of myelin leave axons unprotected and slow down or even block saltatory conduction of electrical signals which contributes to clinical impairment. Moreover, naked axons are highly susceptible to the overall inflammatory environment ultimately resulting in neuronal damage and neurodegeneration, the extent of which dictates the level of permanent neurological disability. Although repair activities are generally limited within the adult CNS, a certain degree of remyelination can be observed. This regenerative process is dependent on successful cell replacement, which is mainly mediated via endogenous oligodendroglial progenitor/precursor cell (OPC) activation [1]. Unfortunately, overall remyelination efficiency remains poor due to limited cellular differentiation and migration [2], [3] or as a consequence of astrocytic cues, such as bone morphogenetic proteins (BMPs), as they have been shown to instruct progenitor cells to differentiate into glial cells expressing astrocyte characteristics [4].

In light of the increasing number of potent immunomodulatory therapies for MS which allow for efficient control of inflammatory relapse activity and preventing further tissue damage [5], the current focus in exploring new MS treatments has shifted towards neuroprotection and functional tissue repair [6]. Regarding the restoration of axonal connectivity and fast signal transmission, recent experimental studies have unravelled a series of distinct molecular switches responsible for the homeostasis and differentiation of stem- and precursor cells within the inflamed tissue [3]. Despite this increasing knowledge on myelinating cell turnover, an integrative translational therapeutic approach aiming at cell-based glial protection and restoration of myelin integrity remains an unmet therapeutic goal. Thus, apart from limiting or re-dressing the deviated immune response, novel methods to support functional regeneration are required.

Mesenchymal stem cells (MSCs) have been shown to exert positive immunomodulatory effects [7], to reduce demyelination [8], to enhance neuroprotection [9] and to promote adult neural stem cell (aNSC) differentiation towards the oligodendroglial lineage at the expense of astrocytes and neurons [10], [11]. Secreted factors remain to be elucidated, with the exception of hepatocyte growth factor (HGF), which has been described as a mediator of recovery in MS models [12]. Moreover, ciliary neurotrophic factor (CNTF) was found to foster stem cell derived oligodendrogenesis but appears not to be an MSC-derived key regulator [13].

Here we investigated to what extent OPCs, as the main cellular source for remyelination, are actively determined and supported in their differentiation process towards myelin building mature oligodendrocytes upon stimulation with soluble mesenchymal factors. We found that MSC-conditioned medium supports oligodendroglial fate decision under astrocytic endorsing conditions enhancing the expression of differentiation markers and promoting morphological maturation. Accelerated maturation featured elevated levels of 2′, 3′-cyclic nucleotide 3′-phosphodiesterase (CNPase) and myelin basic protein (MBP) expression and reduced glial fibrillary acidic protein (GFAP) expression. This process was accompanied by downregulation of prominent inhibitory differentiation factors such as Id2 and Id4 and could not be mimicked by HGF. We thus conclude that apart from their suggested role as a prospective anti-inflammatory MS treatment these cells might also be applied in order to support endogenous myelin repair activities.

## Materials and Methods

### Ethics Statement

All cell preparations and all animal care were carried out in accordance with the European Communities Council Directive (86/609/EEC). Furthermore, we received from the ethics committee of the animal research facility of the Heinrich-Heine-University permits to kill animals and to preserve tissues (O69/2011; O118/11). Ethical considerations and details on the generation of primary oligodendroglial cells as well as of mesenchymal stem cells have previously been reported by us [10], [14]–[16].

### Mesenchymal Stem Cell Preparation and Medium Conditioning

Bone marrow plugs were harvested from femural and tibial bone of 2–4 month-old female Fisher rats (Charles River Deutschland GmbH, Germany). Adult rats were anesthetized using ISOFLURAN (DeltaSelect, Langenfeld, Germany) and killed by decapitation. Plugs were mechanically dissociated in Minimum Essential Medium alpha Medium (α-MEM) (Gibco Cell Culture, Life Technologies, Germany) and recovered by centrifugation. Cell pellets were resuspended in α-MEM containing 10% fetal bovine serum (α-MEM-10%FBS; PAN Biotech GmbH, Germany) and seeded at 1×10^6^ cells/cm^2^ in a humidified incubator at 37°C with 5% CO_2_. After three days, the entire culture was used and designated as bone marrow cells (BMCs) or separated into non-adherent cells and adherent cells. Adherent cells were incubated in fresh **α**-MEM-10%FBS as standard/control medium (designated as α-MEM from here on) until a confluent layer of cells was reached. These cells were trypsinized using a 0.25% Trypsin-EDTA solution (Gibco Cell Culture, Life Technologies, Germany), seeded in α-MEM-10%FBS at 8,000 cells/cm^2^. After 3–5 days of culture, the resulting monolayer of cells, hereafter named rat bone marrow-derived mesenchymal stem cells (MSCs), was trypsinized and frozen or further cultured for experiments. MSC-conditioned medium (MSC-CM) was prepared by plating 12,000 MSCs per cm^2^ and incubation in α-MEM-10% FBS or α-MEM-1% FBS for three days. Conditioned media were not diluted and directly applied to OPCs 4 h to 6 h after plating. HGF (R&D Systems, Minneapolis, MN) was applied at a final concentration of 50 ng/ml in α-MEM-10% FBS according to Bai and colleagues [12]. For HGF neutralization experiments, α-MEM-10% FBS and MSC-CM were incubated at 37°C with a final concentration of 10 µg/ml function-blocking anti-HGF antibody (R&D Systems) for 1 h before use as previously published [12].

### Oligodendroglial Cell Culture

Purification and culturing of OPCs was performed according to earlier descriptions [17]. Briefly, P1 rats were anesthetized using ISOFLURAN and killed by decapitation. Dissociated P1 rat cortices were cultured on poly-D-lysine (PDL)-coated cell culture flasks in DMEM substituted with 10% FBS and 4 mM L-glutamine. After 10 days, flasks were shaken at 250 rev/min for 2 h to deplete from microglial contamination. Then flasks were shaken for another 20 h in which OPCs were dislodged from the underlying astrocyte-layer and replated on PDL-coated culture dishes or glass cover slips in high glucose DMEM-Sato-based medium containing bovine 5 µg/mL insulin, 50 µg/mL human transferrin, 100 µg/mL BSA, 6.2 µg/mL progesterone, 16 µg/mL putrescine, 5 ng/mL sodium selenite, and 4 mM L-glutamine (all Sigma-Aldrich, Missouri, USA). After cell sedimentation (4 to 6 h after plating) the medium was changed after one washing step with PBS to control medium or MSC-CM (no further dilution), which was changed every third day. For morphological staging, OPCs were transfected with a citrine expression vector and morphologies were determined by means of a morphological key as previously described in [14], [15].

### RNA Preparation, cDNA Synthesis and Quantitative Reverse Transcription (RT)-PCR

Prepared OPCs were cultured for up to nine days in α-MEM or MSC-CM. Total RNA purification from cells was done using the RNeasy procedure (Qiagen, Hilden, Germany). Isolated RNA was reverse transcribed using the high capacity cDNA Reverse Transcription Kit (Applied Biosystems, Darmstadt, Germany). Quantitative determination of gene expression levels was performed on a 7900HT sequence detection system (Life Technologies, Applied Biosystems, Darmstadt, Germany) using Power SybrGreen universal master mix (Applied Biosystems). Primer sequences were determined using PrimerExpress 2.0 software (Applied Biosystems) and tested for the generation of specific amplicons: GFAP_fwd: CTG GTG TGG AGT GCC TTC GT, GFAP_rev: CAC CAA CCA GCT TCC GAG AG, CGT_fwd: CCG GCC ACC CTG TCA AT, CGT_rev: CAG GGA GAC GAG TCA CAA CGT, CNPase_fwd: CTG CCG CCG GGA CAT, CNPase_rev: TCC CGC TCG TGG TTG GTA T, MBP_fwd: CAA TGG ACC CGA CAG GAA AC, MBP_rev: TGG CAT CTC CAG CGT GTT C, Id2_fwd: AGA ACC AAA CGT CCA GGA CG, Id2_rev: TGC TGA TGT CCG TGT TCA GG, Id4_fwd: CAG CTG CAG GTC CAG GAT GT, Id4_rev: AAA GTG GAG ATC CTG CAG CAC, GAPDH_fwd: GAA CGG GAA GCT CAC TGG C, GAPDH_rev: GCA TGT CAG ATC CAC AAC GG, ODC_fwd: GGT TCC AGA GGC CAA ACA TC, ODC_rev: GTT GCC ACA TTG ACC GTG AC. GAPDH and ODC were used as reference genes, and relative gene expression levels were determined according to the ΔΔCt method (Applied Biosystems). Each sample was measured in quadruplicate; data are shown as mean values ± SEM and t-test was applied in order to determine statistical significance (Prism 5.0c; GraphPad Software).

### Immunohistochemical Procedures

For marker expression analysis, OPCs were fixed with 4% paraformaldehyde/PBS solution, PBS washed, blocked for 45 minutes using 1% normal goat serum and 1% Triton in PBS and subjected to antibody incubation at 4°C overnight: mouse anti-myelin basic protein (MBP) (1∶500, Sternberger Monoclonals, Lutherville, MD), mouse anti-2′, 3′-cyclic nucleotide 3′-phosphodiesterase (CNPase) (1∶500, Sternberger Monoclonals), rabbit anti-cleaved Caspase3 (1∶500, Cell Signaling Technology, Leiden, The Netherlands), rabbit anti-Ki67 (1∶500, Millipore, Schwalbach, Germany), rabbit anti-GFAP (1∶1000, Dako, Hamburg, Germany), mouse anti-GFAP (1∶1000, Millipore), mouse anti-A2B5 (1∶200, Millipore) and rabbit anti-Olig2 (1∶1000, Millipore). Following PBS washes secondary anti-mouse and anti-rabbit antibodies conjugated with either Alexa Fluor594 or Alexa Fluor488 (1∶1000, Invitrogen) were added for 2 h at room temperature. Nuclei were stained with 4′,6-diamidino-2-phenylindole (Roche). Cells were mounted under Citifluor (Citifluor, Leicester, UK) and analyzed using an Axio Cam HRc microscope (Zeiss, Jena, Germany). HGF-ELISA was performed using Mouse/Rat HGF Quantikine ELISA kit according to the protocol of the supplier (R&D Systems). Absolute cell numbers were determined using the nucleus counter macro of the ImageJ 1.46e software.

### Western Blot Analysis

Lysis of control and MSC-CM treated OPCs was carried out on ice with radioimmu-noprecipitation assay buffer (RIPA buffer; Cell Signaling Technology, Danvers, Massachusetts, USA) with addition of Halt protease inhibitor cocktail (Thermo Scientific, Rockford, IL, USA). Specimens were subjected to two sonification cycles of 15 s each, and protein solutions were kept on ice for 1 min between the pulses. Protein concentrations were determined using the DC Protein Assay (BioRad, München, Germany). Probes were subjected to standard SDS gel electrophoresis and Western blotting using RunBlue SDS gels (Expedeon, Cambridgeshire, UK), RunBlue Blot Sandwich nitrocellulose (Expedeon) applying mouse anti-MBP (MBP; Covance; 1∶500, Princeton, New Jersey, USA), mouse anti-glyceraldehyde-3-phosphate dehydrogenase (GAPDH; Millipore; 1∶1000) antibodies. Signals were visualized using IRDye 680LT donkey anti-mouse and IRDye 800CW donkey anti-mouse antibodies (1∶15000) and an Odyssey infrared imaging system scanner (both LI-COR Biosciences, Lincoln, NE). Protein quantifications were performed using the Odyssey software.

## Results

### Mesenchymal Factors Promote Morphological Maturation of Oligodendroglial Progenitor Cells

**Figure 1:**
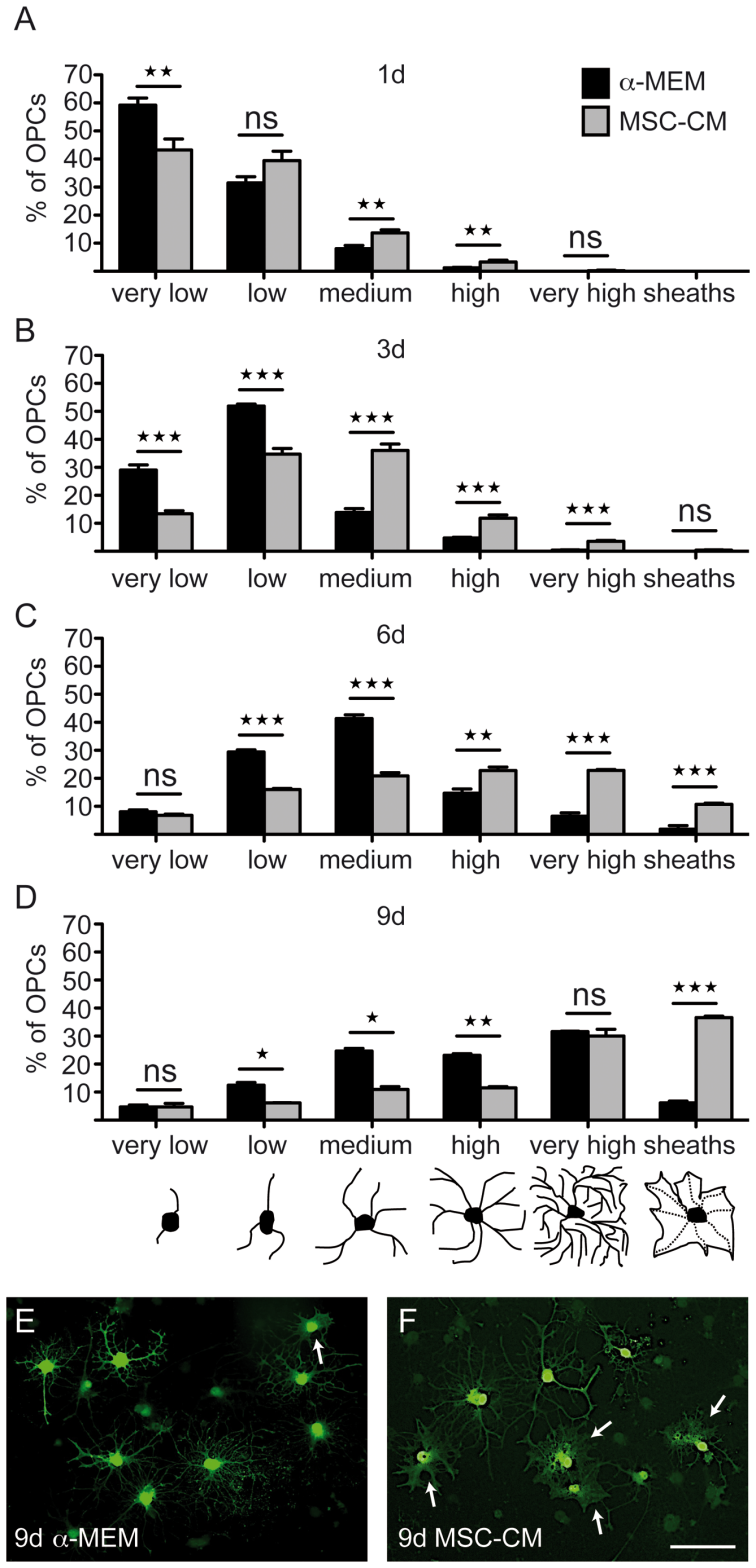
MSC-CM stimulates morphological maturation of OPCs. Mesenchymal stem cell conditioned medium (MSC-CM) accelerates maturation of cultured oligodendroglial progenitors cells. We identified six distinct morphologies (see bottom), from a very low number of processes in progenitor cells to multiple process-bearing cells (low, medium, high) to mature cells with a very high degree of arborisation or flattened appearance (sheaths). (**A–D**) Analysis of OPC morphology distribution revealed an MSC-CM-dependent shift towards more mature cells (black bars **α**-MEM treated cells; grey bars MSC-CM stimulated cells) after one (**A**), three (**B**), six (**C**) and nine days (**D**). (**E,F**) Representative citrine labelled OPCs revealing advanced morphologies and promoted sheath formation (arrows) upon MSC-CM stimulation after nine days. Data are shown as mean values ± SEM derived from n = 5 experiments. t-test (ns: not significant, * P<0.05; ** P<0.01; *** P<0.001). Scale bar: 50 µm.

We have previously shown that co-culture of bone marrow derived MSCs and aNSCs instructs neural stem cells to become oligodendroglial. This process could be successfully mimicked by the application of mesenchymal stem cell conditioned medium [10]. In order to investigate mesenchymal mediated effects on oligodendroglial cells, OPCs were grown in control condition (non-conditioned α-MEM) or mesenchymal stem cell conditioned medium (MSC-CM) and the distribution of cellular morphologies was determined as described previously [14], [15]. We discriminated between early progenitor stages with low numbers of processes (“very low” and “low”), more differentiated cellular stages with increased number of processes and the occurrence of branches (“medium” and “high”), cells with elaborate branching patterns (“very high”) and cells with a flattened appearance featuring sheath formation. Phenotype scoring was performed after one, three, six and nine days of treatment. Absolute numbers of counted cells for all experiments are shown in Table S1. We detected a consistent shift towards the establishment of more complex morphologies at all time points among MSC-CM stimulated cells as compared to control cells (Fig. 1). Of note, at late time points a markedly increased degree of sheath forming cells was detected (Fig. 1D,E,F) indicating that secreted mesenchymal stem cell factors exert a positive effect on oligodendroglial maturation kinetics as well as on process fusion and sheath generation.

### MSC-CM Leads to Increased Levels of Myelin Expression

**Figure 2:**
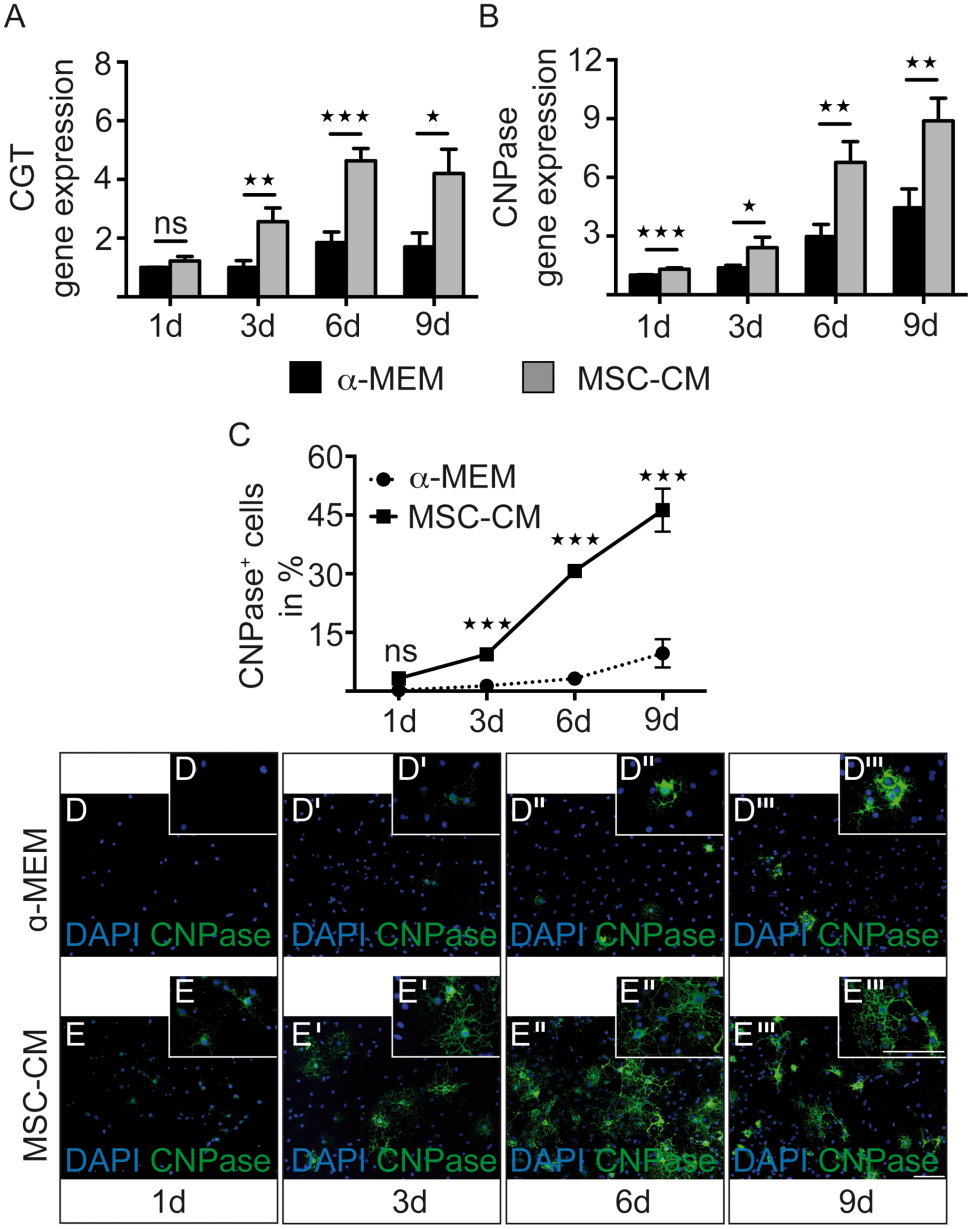
MSC-CM leads to enhanced early myelin expression. Determination of transcript levels by means of quantitative real-time RT-PCR. (**A**) Upregulation of ceramide galactosyltransferase (CGT) expression was detected after three, six and nine days in culture, (**B**) whereas gene expression levels of CNPase were elevated at every measured time point. Glyceraldehyde-3-phosphate dehydrogenase (GAPDH) expression was used as reference. (**C**) In addition, an increased percentage of CNPase-expressing OPCs was observed among MSC-CM treated cells as compared to cells grown in **α**-MEM. Significant differences were detected from day three onwards. (**D–E’’’**) Representative immunofluorescent stainings of CNPase expressing OPCs at all four-time points of investigation. Data are shown as mean values ± SEM and derive from n = 8 (CGT), n = 8 (CNPase, q-RT-PCR) and n = 4 (CNPase, immunostainings) experiments. t-test (* P<0.05; ** P<0.01; *** P<0.001). Scale bars: 50 µm.

A main component of oligodendroglial cell maturation is the expression of myelin markers. We therefore evaluated transcript levels of the ceramide galactosyltransferase (CGT) gene, which encodes an enzyme known to synthesize the myelin component galactosylceramide (GalC). CGT was significantly increased by 3-fold in OPCs after MSC-CM treatment for three days and by 2.5-fold after six and nine days compared to control (Fig. 2A). Additionally, transcript levels of the early myelin marker CNPase were induced from one to nine days of treatment (Fig. 2B). To evaluate, whether CNPase protein levels were also elevated, we stained and determined the percentage of CNPase positive cells in both conditions. This demonstrated that in OPC populations treated with MSC-CM, CNPase protein expression is strongly and significantly increased at time points three, six and nine days after stimulation (Fig. 2C–E’’’).

**Figure 3:**
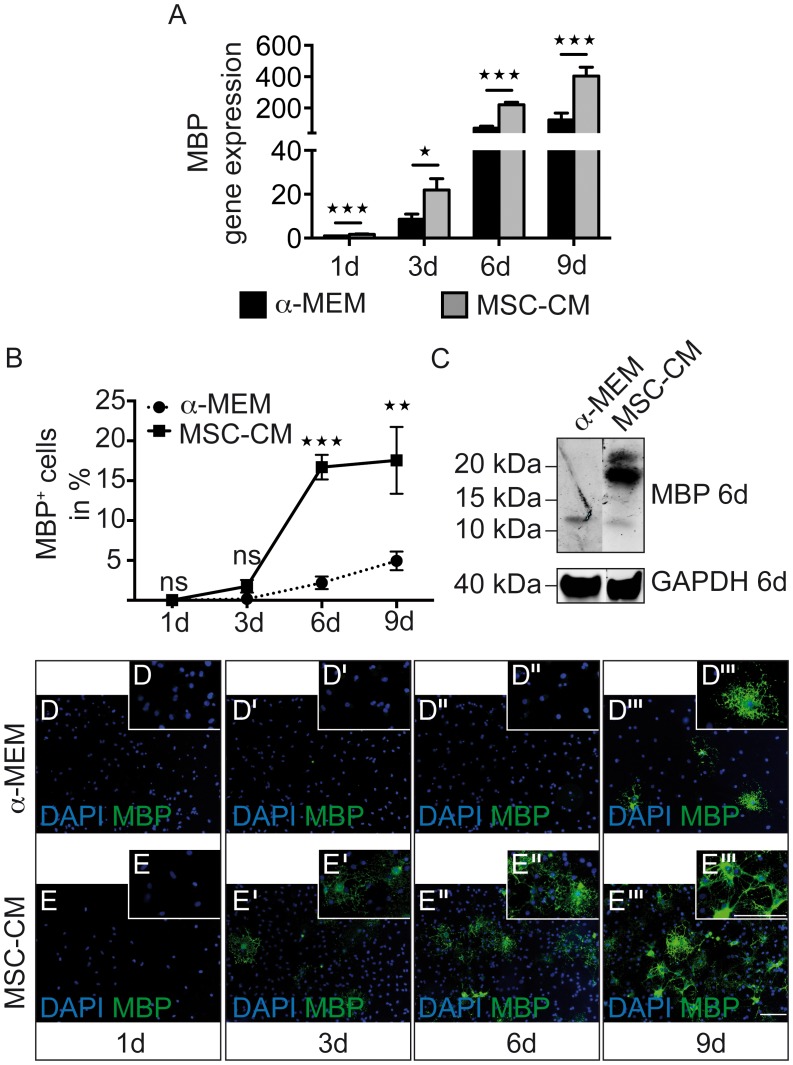
MSC-CM enhances myelin basic protein expression. (**A**) Gene expression levels of MBP are up-regulated after one, three, six and nine days in MSC-CM. (**B**) Determination of the degree of MBP-positive OPCs as revealed by immunostainings show a significantly increased number after MSC-CM treatment for six and nine days. (**C**) Western blot analysis shows increased MBP protein levels after six days in mesenchymal stem cell conditioned medium. GAPDH was used as internal reference. (**D–E’’’**) Representative immunofluorescent stainings of MBP expressing OPCs at all four time points of investigation. Data are shown as mean values ± SEM derived from n = 6 (q-RT-PCR) and n = 3 (immunostainings, Western Blot) experiments. t-test (* P<0.05; ** P<0.01, *** P<0.001). Scale bars: 50 µm.

**Figure 4:**
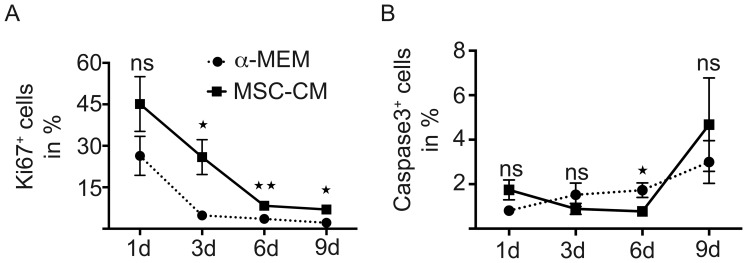
MSC-CM treatment enhances OPC proliferation, while cell death rate remains low. (**A**) Anti-Ki67 immunofluorescent stainings and evaluation of positive cells revealed a higher proliferation rate under MSC-CM treatment as compared to **α**-MEM, whereas (**B**) cell death rates remained low under both conditions with a minor significant survival effect upon MSC-CM treatment at six days. Data are shown as mean values ± SEM derived from n = 4 experiments. t-test (ns: not significant, * P<0.05; **P<0.01).

Likewise the degree of MBP-expressing OPCs was elevated with significant changes on gene expression levels at every time-point (Fig. 3A), and after six and nine days of treatment for protein levels (Fig. 3B,D–E’’’) as well as in Western blot analysis (Fig. 3C). To confirm that these observed effects were not based on selection processes, we examined proliferation and cell death of control medium and MSC-CM treated OPCs. During the culture of OPCs, proliferation rates gradually decreased but were slightly increased in the presence of mesenchymal secreted factors with significant alterations found after three, six and nine days (Fig. 4A). The percentage of Caspase3-positive OPCs between the two conditions was only altered significantly on day six, though the overall Caspase3-positivity remained low (Fig. 4B).

**Figure 5:**
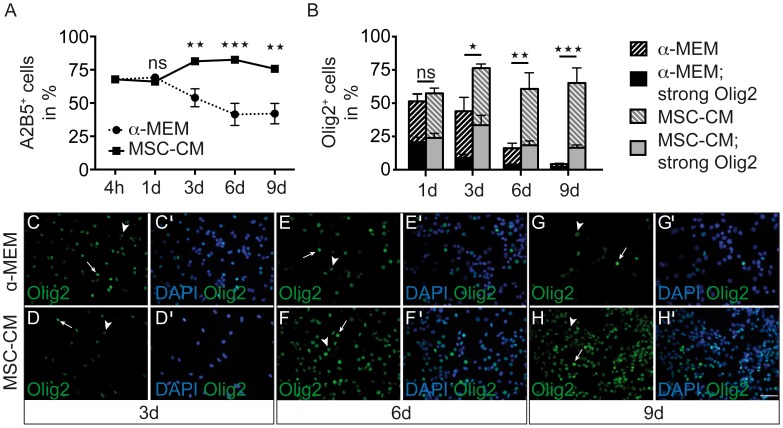
MSC-CM dependent regulation of early OPC markers. (**A**) Determination of the degree of A2B5 positive cells as revealed by immunofluorescence stainings. **α**-MEM and MSC-CM were given to the cells after four hours. (**B**) Determination of the degree of weak and strong Olig2 expressing cells during the course of **α**-MEM and MSC-CM stimulation up to nine days. (**C–H’**) Representative immunofluorescent stainings of Olig2 expressing OPCs at all three time points. Arrowheads mark weak expressing cells, whereas arrows point to strong expressors. Data are shown as mean values ± SEM derived from n = 3 (A2B5) and n = 4 (Olig2) experiments. t-test (ns: not significant, * P<0.05; **P<0.01; *** P<0.001). Scale bar: 50 µm.

In order to determine, whether mesenchymal stem cell-derived factors affect oligodendroglial fate decision and stability, we investigated expression of early markers such as A2B5 and Olig2. These experiments confirmed that the majority of plated cells are A2B5 positive and that the expression is decreased under **α**-MEM conditions whereas it is increased upon MSC-CM treatment (Fig. 5A). Likewise, MSC-CM treatment increased the percentage of both weak as well as of strong Olig2 expressors as opposed to strongly decreased levels under **α**-MEM conditions (Fig. 5B–H’).

### Regulation of Astroglial Factors

**Figure 6:**
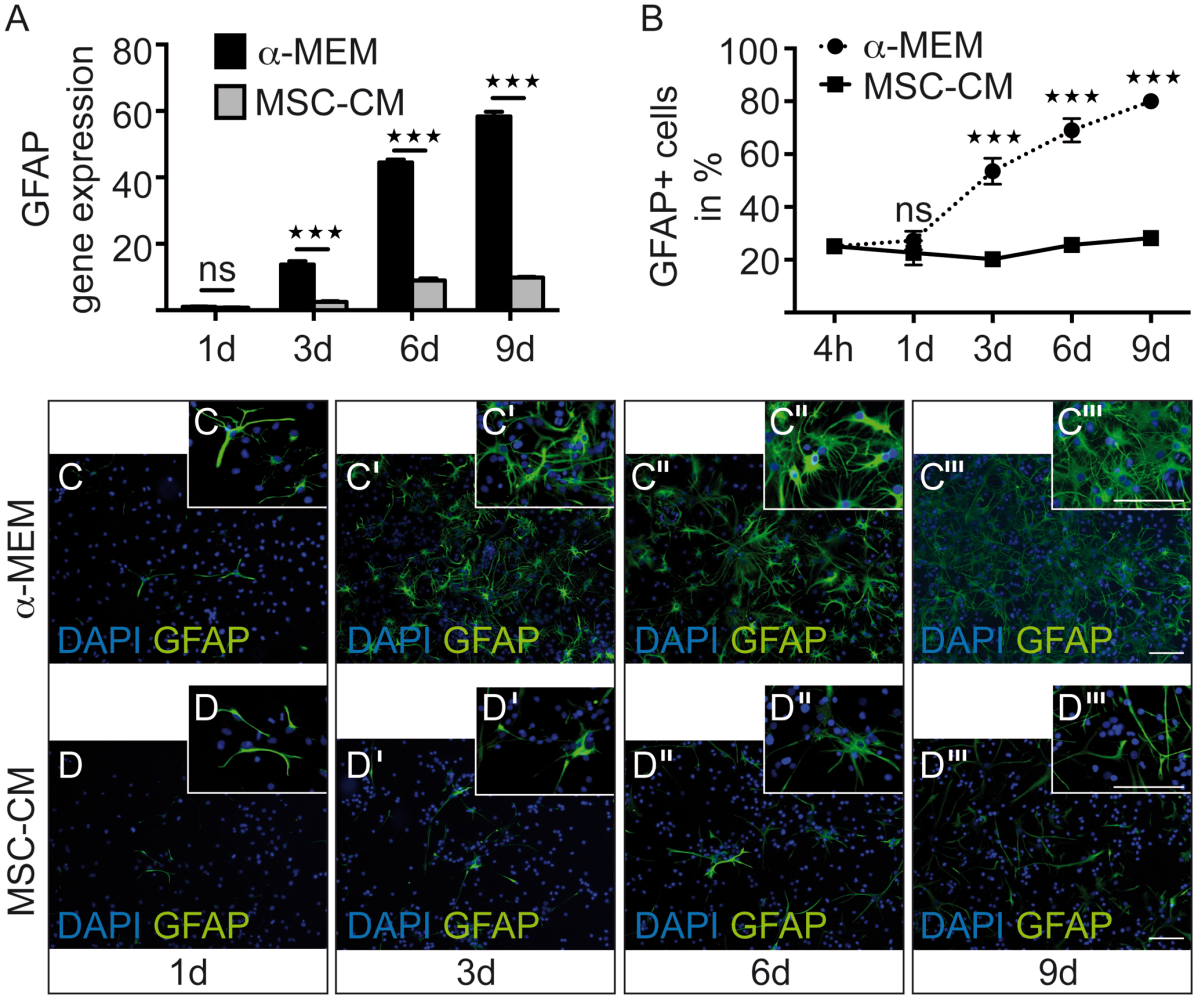
Downregulation of GFAP expression following stimulation with MSC-CM. OPCs were plated for four hours in SATO medium before changing the medium to control (**α**-MEM) or to mesenchymal stem cell conditioned medium (MSC-CM) and analysis after one, three, six and nine days. (**A**) Consistently decreased GFAP transcript levels were detected at every time point using quantitative real-time RT-PCR. (**B**) Determination of the degree of GFAP-positive OPCs revealed increasing numbers among **α**-MEM treated cells whereas MSC-CM stimulation stabilized low GFAP expression levels. (**C–D’’’**) Representative anti-GFAP immunofluorescent stainings. Data are shown as mean values ± SEM derived from n = 3 for both, q-RT-PCR as well as immunostaining experiments. t-test (*** P<0.001). Scale bars: 50 µm.

**Figure 7:**
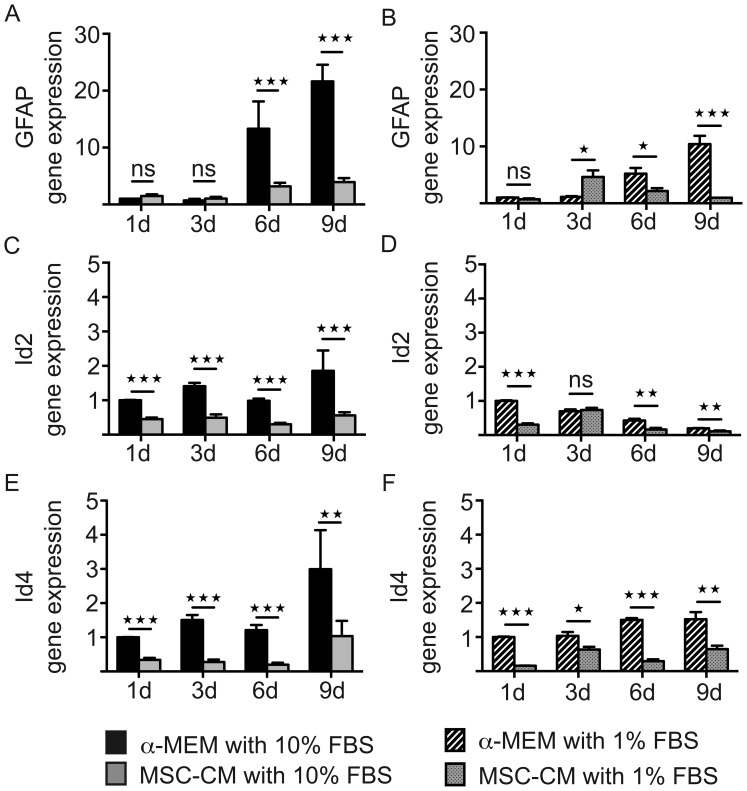
Serum reduced conditioned medium. Determination of transcript levels by means of quantitative real-time RT-PCR. Investigation of GFAP, Id2 and Id4 gene expression levels in **α**-MEM versus MSC-CM containing 10% FBS (**A,C,E**). Investigation of GFAP, Id2 and Id4 gene expression levels in **α**-MEM versus MSC-CM containing 1% FBS (**B,D,F**). Glyceraldehyde-3-phosphate dehydrogenase (GAPDH) expression was used as reference gene; data are shown as mean values ± SEM derived from n = 7 for Id2, Id4 and n = 3 for GFAP (**A,C,E**) and n = 3 (**B,D,F**) experiments. t-test (ns: not significant, * P<0.05; **P<0.01; *** P<0.001).

We next analyzed, whether the astrocyte marker glial fibrillary acidic protein (GFAP) was regulated by MSC-CM. Indeed, OPCs incubated with MSC-CM displayed strongly reduced GFAP transcript levels and a significantly lowered degree of GFAP positivity (Fig. 6). This was in contrast to cells grown in **α**-MEM featuring a gradual increase in GFAP protein expression over time, thereby accumulating astrocytic features and gaining astrocytic morphologies. In accordance to Ki67 and Caspase-3 data presented above (Fig. 4), we conclude that the increase of GFAP positive cells in **α**-MEM cannot derive from either increased proliferation or promoted survival of astrocytic cells. Moreover, given the known astrogliogenic potential of high serum containing media, we generated MSC-CM based on **α**-MEM containing 1% FBS only (instead of 10% FBS as shown in Fig. 6). We directly compared gene expression levels in high an low serum containing media and observed that although the astrogliogenic pressure is lower under serum-reduced conditions, MSC-CM can still efficiently counteract the induction of astrocytic features. However, the extent and temporal profiles of GFAP expression revealed to be slightly different (Fig. 7A compare to Fig. 7B).

Initially described as O-2A cells [18], OPCs exert a certain degree of multipotency which under particular circumstances can also give rise to astrocytes [19]. Promotion of astrogliosis has also been reported for the **α**-MEM medium which was applied in this study in particular due to the presence of fetal bovine serum (FBS) [10], [20], [21]. Furthermore, transcriptional regulators such as inhibitor of differentiation 2 (Id2) and inhibitor of differentiation 4 (Id4) have been described to control glial differentiation acting also as inhibitors of myelin expression [22], [23]. We therefore examined whether MSC-CM plays a role in fate decision of immature OPCs via regulation of Id2/4 expression levels and detected significantly decreased Id2 an Id4 transcript levels in OPCs upon MSC-CM stimulation at all time points (Fig. 7C,E). Of note, similar Id regulations were observed when low serum MSC-CM was applied (Fig. 7D,F). However, due to the lowered astrogliogenic potential of serum reduced medium, gene expression levels were lower as compared to the 10% FBS containing media.

### Hepatocyte Growth Factor as a Secreted MSC-CM Factor is not the Critical Component for OPC Differentiation

**Figure 8:**
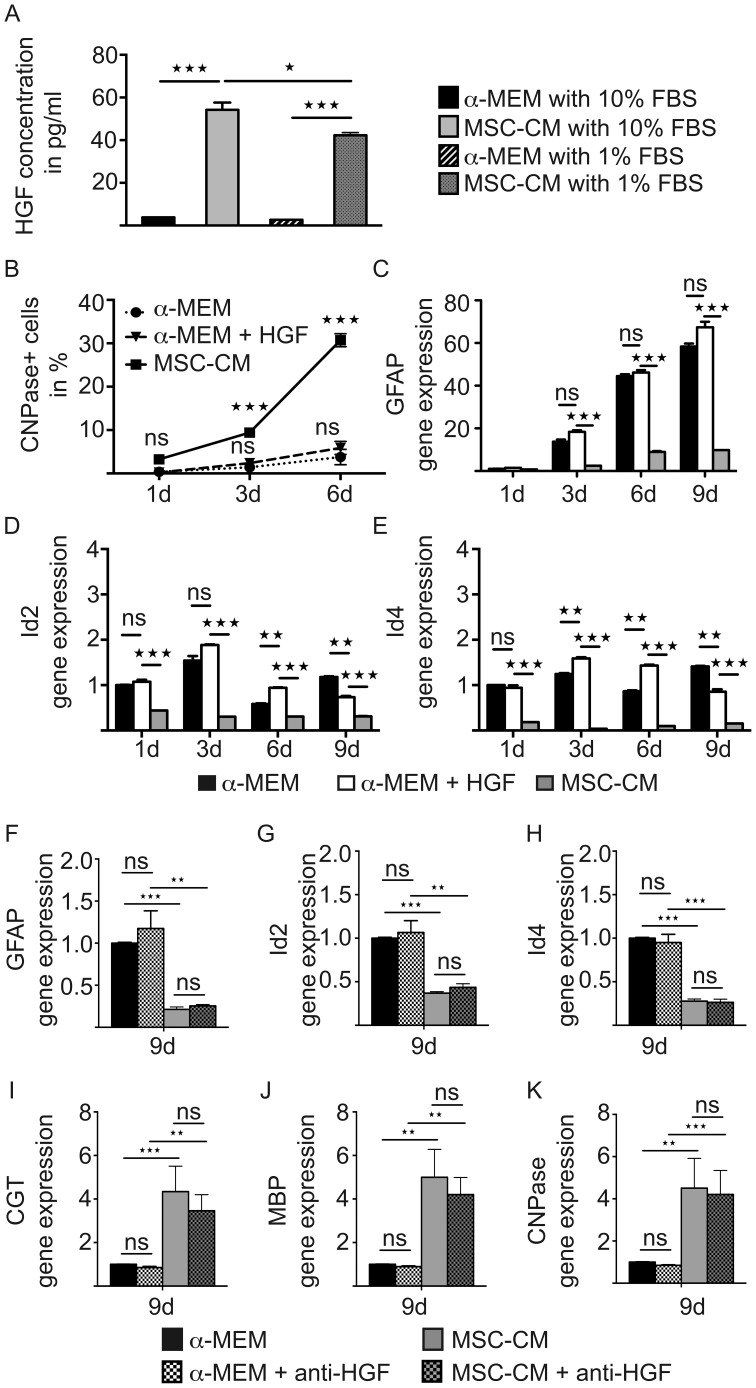
HGF does not mimic MSC-CM mediated cellular effects. (**A**) Determination of HGF protein content in stimulation media by means of ELISA revealing a robust increase upon MSC conditioning. (**B**) No difference regarding CNPase positivity was observed among OPCs grown in **α**-MEM in the absence or presence of recombinant HGF, whereas MSC-CM reproducibly increased CNPase expression. (**C–E**) Determination of transcript levels by means of quantitative real-time RT-PCR. No significant differences regarding Id2, Id4 and GFAP transcript levels were observed among OPCs grown in α-MEM in the absence or presence of recombinant HGF. MSC-CM treatment significantly reduced transcript levels of all three genes at all time points. (**F–K**) Anti-HGF antibody mediated depletion experiments revealed no effect on astrocyte (GFAP, Id2, Id4) and oligodendroglial/myelin (CGT, MBP, CNPase) gene expression levels. Glyceraldehyde-3-phosphate dehydrogenase (GAPDH) expression was used as reference gene. All data are shown as mean values ± SEM derived from n = 3 experiments for each analysis. t-test (ns: not significant, * P<0.05; ** P<0.01; *** P<0.001).

The composition of mesenchymal stem cell secreted factors and their molecular interactions are still poorly understood. Among the few identified molecules is hepatocyte growth factor (HGF), which has been shown to mediate mesenchymal stem cell-induced recovery in animal MS models [12]. We therefore evaluated whether HGF is also a key regulator of the described MSC-CM effect exerted on immature OPCs. To this end, we determined HGF content using enzyme linked immunosorbent assay (ELISA) revealing a significant enrichment of HGF in MSC-CM after a three days incubation of MSCs with **α**-MEM containing either 10% or 1% FBS (Fig. 8A). To clarify, whether HGF exerts an oligodendroglial differentiation effect, we quantified CNPase-positive cells in **α**-MEM supplemented with 50 ng/ml recombinant HGF. Importantly, we did not observe any significant differences between **α**-MEM cultured with or without HGF whereas both conditions induced significantly lower CNPase expression levels compared to MSC-CM (Fig. 8B). Moreover, GFAP expression levels of cells grown in **α**-MEM plus HGF were similar to those kept in **α**-MEM (Fig. 8C). Likewise, Id2/4 transcript levels were also not down-regulated by HFG, as compared to **α**-MEM grown cells, and were even further increased after three and six days in culture (Fig. 8D,E). We also analyzed gene expression levels after neutralizing HGF with a function-blocking antibody based on previously published conditions [12]. We analyzed GFAP, Id2, Id4, CGT, MBP and CNPase gene expression levels at time-point nine days of stimulation. Importantly, we did not detect any differences between media without and media with anti-HGF antibodies (Fig. 8F–K), further supporting that HGF is not involved in the MSC-CM dependent fate and differentiation effect.

## Discussion

Oligodendroglial cell maturation is coordinated by different factors either stimulating or inhibiting differentiation processes. Here, we demonstrate that mesenchymal stem cell secreted factors of yet unknown identity support and enforce cell fate decisions and promote differentiation and maturation towards oligodendrocytes. Furthermore, a slight MSC-CM mediated increase in OPC proliferation was observed which is in line with previous data on the control of neural stem cell proliferation [24].

The observation that OPCs did not develop astrocytic features and morphologies in the presence of mesenchymal stem cell-secreted components leads to the assumption that cell fate decisions are regulated. Although currently unidentified, this mixture of factors apparently influences glial transcription patterns – some of the regulated genes being dependent on Olig2 and Id-modulated bHLH transcriptional activities. In this regard, the regulation of Id2 and Id4 is most noteworthy as, for the first time, it demonstrates a glial cell regulatory effect based on mesenchymal stem cells independent of FBS concentration in the medium used for conditioning. Since adding as well as neutralizing HGF turned out to be ineffective in either reversing the fate of fetal bovine serum containing medium instructed cells or in supporting oligodendroglial differentiation, future investigations will have to address the nature of the underlying conditioning factors. It is likely that it will be a cocktail of different ingredients that proves to be essential with perhaps different combinations acting on fate decisions while others influence particular maturation steps. Moreover, the finding that HGF is not the responsible trophic substance is in agreement with previous findings on an HGF-dependent blockade under oligodendroglial differentiation permissive conditions [25]. Of note, we have previously described that another MSC-derived factor, ciliary neurotrophic factor (CNTF), is also not part of the soluble activity acting on aNSCs [13].

The finding of an MSC-CM-dependent counteracting of astrocytic cues leads to the hypothesis that mesenchymal stem cells have the potential to also activate resident stem- and precursor cells upon therapeutic transplantation (locally or systemically). In light of the fact that in contrast to niche-derived NSCs, resident OPCs represent a widespread and dispersed cell population in the CNS promotion of endogenous repair activities by activated and fate-stabilized OPCs provides important advantages. Together with the proliferative enhancement of early progenitor cells and in addition to their proven immunomodulatory effects, these features could indeed contribute to successful myelin repair *in vivo*. Of note, bone morphogenetic protein (BMP) signalling leading to increased Id4 and decreased Olig2 expression has been shown to cause astrocyte differentiation of OPCs [26]. Additionally, after demyelinating injuries, BMPs were shown to promote gliosis [27] and subsequent glial scar formation which interferes with axonal restoration as well as remyelination. Our findings therefore also suggest that MSC-CM pre-treated exogenous OPCs could be a valuable tool for functional exogenous cell replacement helping to withstand astrocytic lesion cues.

Lindsay and colleagues recently demonstrated that conditioning effects may differ between MSCs derived from different tissues, with human mesenchymal stem cells from the lamina propria having the ability to enhance *in vitro* myelination whereas bone marrow-derived cells failed to do so [28]. However, their study focused on oligodendroglial cells which had been cultured for seven days in medium supplemented with growth factors and which are therefore most likely more advanced in terms of fate stabilisation and maturation as well as on olfactory ensheathing cells (OECs). Nevertheless, this study suggests that mesenchymal cocktails are origin-specific, a notion that might be experimentally explored in the future for the identification of active components.

In conclusion, our study provides strong evidence for cell fate decision- as well as differentiation promoting properties of secreted mesenchymal stem cell factors acting on OPCs. As these resident progenitor cells constitute the major source for myelinating glial cell replacement in demyelinating CNS diseases, stimulation of endogenous repair activity by means of MSC transplantation or administration of responsible underlying trophic factors represents an attractive therapeutic approach.

## Supporting Information

Table S1 **Absolute numbers of counted cells in this study.** For each counting at least three different experiments per condition were analyzed.
(XLSX)

## References

[1] Chang A, Nishiyama A, Peterson J, Prineas J, Trapp BD (2000) NG2-positive oligodendrocyte progenitor cells in adult human brain and multiple sclerosis lesions. J Neurosci 20: 6404–6412. 10964946 10.1523/JNEUROSCI.20-17-06404.2000 PMC6772992

[2] Kremer D, Aktas O, Hartung HP, Küry P (2011) The complex world of oligodendroglial differentiation inhibitors. Ann Neurol 69: 602–618. 21520230 10.1002/ana.22415

[3] Kotter MR, Stadelmann C, Hartung HP (2011) Enhancing remyelination in disease–can we wrap it up? Brain 134: 1882–1900. 21507994 10.1093/brain/awr014

[4] Mabie PC, Mehler MF, Marmur R, Papavasiliou A, Song Q, et al (1997) Bone morphogenetic proteins induce astroglial differentiation of oligodendroglial-astroglial progenitor cells. J Neurosci 17: 4112–4120. 9151728 10.1523/JNEUROSCI.17-11-04112.1997 PMC6573548

[5] Aktas O, Kieseier B, Hartung HP (2010) Neuroprotection, regeneration and immunomodulation: broadening the therapeutic repertoire in multiple sclerosis. Trends Neurosci 33: 140–152. 20045200 10.1016/j.tins.2009.12.002

[6] Mullard A (2011) Success of immunomodulators in MS shifts discovery focus to neuroprotection. Nat Rev Drug Discov 10: 885–887. 22129976 10.1038/nrd3610

[7] Aggarwal S, Pittenger MF (2005) Human mesenchymal stem cells modulate allogeneic immune cell responses. Blood 105: 1815–1822. 15494428 10.1182/blood-2004-04-1559

[8] Gordon D, Pavlovska G, Uney JB, Wraith DC, Scolding NJ (2010) Human mesenchymal stem cells infiltrate the spinal cord, reduce demyelination, and localize to white matter lesions in experimental autoimmune encephalomyelitis. J Neuropathol Exp Neurol 69: 1087–1095. 20940628 10.1097/NEN.0b013e3181f97392

[9] Uccelli A, Benvenuto F, Laroni A, Giunti D (2011) Neuroprotective features of mesenchymal stem cells. Best Pract Res Clin Haematol 24: 59–64. 21396593 10.1016/j.beha.2011.01.004

[10] Rivera FJ, Couillard-Despres S, Pedre X, Ploetz S, Caioni M, et al (2006) Mesenchymal stem cells instruct oligodendrogenic fate decision on adult neural stem cells. Stem Cells 24: 2209–2219. 16763198 10.1634/stemcells.2005-0614

[11] Bai L, Lennon DP, Eaton V, Maier K, Caplan AI, et al (2009) Human bone marrow-derived mesenchymal stem cells induce Th2-polarized immune response and promote endogenous repair in animal models of multiple sclerosis. Glia 57: 1192–1203. 19191336 10.1002/glia.20841 PMC2706928

[12] Bai L, Lennon DP, Caplan AI, DeChant A, Hecker J, et al (2012) Hepatocyte growth factor mediates mesenchymal stem cell-induced recovery in multiple sclerosis models. Nat Neurosci 15: 862–870. 22610068 10.1038/nn.3109 PMC3427471

[13] Rivera FJ, Kandasamy M, Couillard-Despres S, Caioni M, Sanchez R, et al (2008) Oligodendrogenesis of adult neural progenitors: differential effects of ciliary neurotrophic factor and mesenchymal stem cell derived factors. J Neurochem 107: 832–843. 18786165 10.1111/j.1471-4159.2008.05674.x

[14] Kremer D, Heinen A, Jadasz JJ, Göttle P, Zimmermann K, et al (2009) p57kip2 is dynamically regulated in experimental autoimmune encephalomyelitis and interferes with oligodendroglial maturation. Proc Natl Acad Sci U S A 106: 9087–9092. 19458044 10.1073/pnas.0900204106 PMC2690052

[15] Göttle P, Kremer D, Jander S, Ödemis V, Engele J, et al (2010) Activation of CXCR7 receptor promotes oligodendroglial cell maturation. Ann Neurol 68: 915–924. 21154415 10.1002/ana.22214

[16] Steffenhagen C, Dechant FX, Oberbauer E, Furtner T, Weidner N, et al (2012) Mesenchymal stem cells prime proliferating adult neural progenitors toward an oligodendrocyte fate. Stem Cells Dev 21: 1838–1851. 22074360 10.1089/scd.2011.0137 PMC3396148

[17] McCarthy KD, de Vellis J (1980) Preparation of separate astroglial and oligodendroglial cell cultures from rat cerebral tissue. J Cell Biol 85: 890–902. 6248568 10.1083/jcb.85.3.890 PMC2111442

[18] Raff MC, Miller RH, Noble M (1983) A glial progenitor cell that develops in vitro into an astrocyte or an oligodendrocyte depending on culture medium. Nature 303: 390–396. 6304520 10.1038/303390a0

[19] See J, Mamontov P, Ahn K, Wine-Lee L, Crenshaw EB (2007) BMP signaling mutant mice exhibit glial cell maturation defects. Mol Cell Neurosci 35: 171–182. 17391983 10.1016/j.mcn.2007.02.012 PMC1950488

[20] Jadasz JJ, Rivera FJ, Taubert A, Kandasamy M, Sandner B, et al (2012) p57kip2 regulates glial fate decision in adult neural stem cells. Development 139: 3306–3315. 22874918 10.1242/dev.074518

[21] Gard AL, Williams WC 2nd, Burrell MR (1995) Oligodendroblasts distinguished from O-2A glial progenitors by surface phenotype (O4+GalC-) and response to cytokines using signal transducer LIFR beta. Dev Biol 167: 596–608. 7875381 10.1006/dbio.1995.1051

[22] Kondo T, Raff M (2000) The Id4 HLH protein and the timing of oligodendrocyte differentiation. EMBO J 19: 1998–2007. 10790366 10.1093/emboj/19.9.1998 PMC305683

[23] Wang S, Sdrulla A, Johnson JE, Yokota Y, Barres BA (2001) A role for the helix-loop-helix protein Id2 in the control of oligodendrocyte development. Neuron 29: 603–614. 11301021 10.1016/s0896-6273(01)00237-9

[24] Yoo SW, Kim SS, Lee SY, Lee HS, Kim HS, et al (2008) Mesenchymal stem cells promote proliferation of endogenous neural stem cells and survival of newborn cells in a rat stroke model. Exp Mol Med 40: 387–397. 18779651 10.3858/emm.2008.40.4.387 PMC2679267

[25] Ohya W, Funakoshi H, Kurosawa T, Nakamura T (2007) Hepatocyte growth factor (HGF) promotes oligodendrocyte progenitor cell proliferation and inhibits its differentiation during postnatal development in the rat. Brain Res 1147: 51–65. 17382307 10.1016/j.brainres.2007.02.045

[26] Cheng X, Wang Y, He Q, Qiu M, Whittemore SR, et al (2007) Bone morphogenetic protein signaling and olig1/2 interact to regulate the differentiation and maturation of adult oligodendrocyte precursor cells. Stem Cells 25: 3204–3214. 17872503 10.1634/stemcells.2007-0284 PMC2742907

[27] Setoguchi T, Yone K, Matsuoka E, Takenouchi H, Nakashima K, et al (2001) Traumatic injury-induced BMP7 expression in the adult rat spinal cord. Brain Res 921: 219–225. 11720729 10.1016/s0006-8993(01)03123-7

[28] Lindsay SL, Johnstone SA, Mountford JC, Sheikh S, Allan DB, et al (2013) Human mesenchymal stem cells isolated from olfactory biopsies but not bone enhance CNS myelination in vitro. Glia 61: 368–382. 23281012 10.1002/glia.22440

